# COVID-19 and Comorbid Hypertension: Is ACE2 the Culprit?

**DOI:** 10.1017/S1049023X20001090

**Published:** 2020-08-25

**Authors:** Ting Zhang, Sen Zhong, Wenzhai Cao

**Affiliations:** 1. PhD of Infectious Diseases, Department of Infectious Diseases, Hospital of Chengdu University of Traditional Chinese Medicine, Chengdu, PR China; 2. Professor, Department of Infectious Diseases, Hospital of Chengdu University of Traditional Chinese Medicine, Chengdu, PR China; 3. Assistant Professor, Department of Cardiology, Zigong First People’s Hospital, Zigong, PR China

**Keywords:** angiotensin-converting enzyme 2, COVID-19, hypertension, SARS-CoV-2

Dear Editor,

A novel coronavirus, the Severe Acute Respiratory Syndrome Coronavirus-2 (SARS-CoV-2), which was discovered in clusters of patients with severe pneumonia in Wuhan, China, has spread rapidly across the continents and has been a global pandemic threat to mankind. Research in China^1^ and Italy^2^ has shown that there is a potential association between comorbid hypertension and the SARS-CoV-2 infection, as well as worse outcomes. A better understanding of the association between hypertension and COVID-19 infection is required to inform better therapeutics and preventive interventions.

SARS-CoV-2 binds of the spike protein (S-protein) through angiotensin-converting enzyme 2 (ACE2), then enters the host cells and triggers infection; ACE2 expression may be associated with viral loads as well as the vulnerability of COVID-19 infection.^3^ A previous study indicated that ACE2 gene expression, as well as ACE2 activity, would be upregulated by the intake of renin-angiotensin-aldosterone system inhibitors.^4^ Based on the hypothesis above, hypertension patients who intake angiotensin converting enzyme inhibitors (ACEIs)/angiotensin receptor blockers (ARBs) may predispose themselves to a susceptibility of SARS-CoV-2.

There are still some doubts about this hypothesis. First, whether higher ACE2 expression of hypertension individuals could be caused by taking ACEIs/ARBs. The latest study reviewing 12 animal experiments and 12 human studies^5^ evaluated the impacts of administration of ACEIs/ARBs on ACE2 level. The higher ACE2 expression reports predominantly occurred in animal studies, in acute injury models, and/or with higher doses of ACEIs or ARBs. Data from human studies overwhelmingly indicated utilization of ACEI/ARBs did not increase ACE2 levels, especially the expression in the lung tissues. Second, could a mild or moderate ACE2 deficiency protect individuals from viral invasion? Due to the intrinsically high affinity of SARS-CoV-2, which is 10-20 times higher than that of previous SARS-CoV, the ACE2 deficiency could not protect from viral invasion. In contrast, at lung level of COVID-19 infection, ACE2 deficiency would facilitate the pulmonary inflammatory lesions, eventually leading to the fatal complication of Acute Respiratory Distress Syndrome (ARDS). Therefore, the down regulation of ACE2 due to viral invasion would be especially harmful to individuals with baseline ACE2 deficiency induced by, for example, older age, diabetes, or hypertension. Finally, the association between ACE2 levels and COVID-19 infection are still uncertain.

Besides the ACE2 level, the ACE2 structural variation and gene polymorphism should also be noted. Some structural variations of human ACE2 have been identified, characterized by a low binding affinity with S-proteins with potential protective implications. Moreover, various ACE2 gene polymorphisms may link to SARS-CoV-2 infections from viral entrance to replication. Heterozygote loss of ACE2 had been proven to increase the susceptibility to heart disease and diabetes mellitus. There was noteworthy minor allele difference in four missense mutations of ACE2 between Asians and Caucasians, two of whom (K26R and I468V) might influence the combine characteristics between S-protein of SARS-CoV-2 and human ACE2 receptor.^6^

Furthermore, apart from the ACE2 expression itself, SARS-CoV-2 needs process-related genes and activation of metabolic pathways for viral replication. Transmembrane protease, serine 2 (TMPRSS2) cleaves the C-terminal fragment of ACE2 and promotes the S-protein to allow cellular uptake. The significance of TMPRSS2 is approved by the evidence that entry of SARS-CoV-2 into cells is partially blocked by camostat mesylate, an inhibitor of TMPSRR2.^7^ The latest research indicated the expression patterns of TMPRSS2 and ACE2 in human myocardial and testicular samples were different and inconsistent by single RNA-seq.^8,9^ These results suggest there may be interactions between ACE2 expression cells and TMPRSS2 expression cells. Ziegler found that there are ACE2 and TMPRSS2 co-expression cells, a rare subset of epithelial cells, in respiratory tissues.^10^ Whether ACE2 and TMPRSS2 are needed on the same cell or some proteases could activate SARS-CoV-2 S-protein to invade ACE2 single-positive cells is still unclear. Therefore, as for the susceptibility of COVID-19 infections with hypertension patients, attention should also be paid to ACE2 and TMPRSS2 expression in the lung tissue and the interaction between them in order to make a comprehensive judgment.

In the future, single-cell sequencing-based technologies will be applied to study the expression level of ACE2 in alveolar type-2 cells of COVID-19 patients with hypertension with different stages of infection, as well as the association between ACEI drugs and ACE2, so as to clarify the role of ACE2 in COVID-19.
